# Perceptions related to breastfeeding and the early introduction of complementary foods amongst migrants in Cape Town, South Africa

**DOI:** 10.1186/s13006-016-0088-3

**Published:** 2016-10-20

**Authors:** Jo Hunter-Adams, Landon Myer, Hanna-Andrea Rother

**Affiliations:** 1 Health Economics Unit, School of Public Health and Family Medicine, Faculty of Health Sciences, University of Cape Town Observatory, Cape Town, 7925 South Africa; 2 Centre for Infectious Disease Epidemiology and Research, School of Public Health and Family Medicine, Faculty of Health Sciences, University of Cape Town Observatory, Cape Town, 7925 South Africa; 3 Environmental Health Division, School of Public Health and Family Medicine, Faculty of Health Sciences, University of Cape Town Observatory, Cape Town, 7925 South Africa

**Keywords:** Breastfeeding, Refugee and immigrant health, South Africa, Infant nutrition, Complementary feeding

## Abstract

**Background:**

Infant feeding recommendations are of health importance, yet the extent to which migrant communities in low- and middle-income countries know or implement these recommendations is poorly understood. This study explores the perspectives of infant feeding amongst cross-border migrants in Cape Town, South Africa.

**Methods:**

Between February and October 2013, semi-structured in-depth interviews (*n* = 23) were conducted face-to-face with Congolese, Somali and Zimbabwean mothers living in Cape Town. To assess commonly identified narratives of infant feeding, nine focus group discussions (three with men and six with women) were conducted with migrant Somalis, Congolese, and Zimbabweans.

**Results:**

Three dominant themes framed infant feeding. 1) Pragmatism in feeding choices drove responses to baby’s cues, including cries, sleeping patterns, and weight gain (2). Formula feeding was normative in the South African context, whereas lack of commercial infant milk back home was described in terms of expense (3). Low rates of breastfeeding were explained in terms of work responsibilities including household work and lack of breastmilk supply resulting from stress and poor diet. However, women participants typically did not consider their feeding choices to negatively affect their baby’s health.

**Conclusions:**

The reasons for early introduction of both commercial infant milk and solid foods were complex. Breastfeeding was not prioritized despite an awareness of medical recommendations. Rather than emphasizing specific breastfeeding intentions, participants favoured an approach that reacted to their baby’s perceived changing needs. The practical challenges of breastfeeding described by cross-border migrant women reflect one way in which socio-economic and health inequalities may currently be perpetuated for marginalised populations.

## Background

In this paper we explore the infant feeding discourses amongst three cross-border migrant populations in Cape Town. We ask: how do migrants navigate decisions related to breastfeeding and complementary feeding? We investigate the motivations underlying current breastfeeding practices amongst this population, and consider how these practices relate to World Health Organization (WHO) feeding recommendations.

WHO breastfeeding recommendations include commencement of breastfeeding within one hour of birth, exclusive breastfeeding until 6 months and continued breastfeeding until 2 years of age or older [1]. Complementary feeding refers to the transition from exclusive breastfeeding to family foods, usually between 6 and 18−24 months of age. The adequacy of complementary feeding is framed in terms of timely, adequate, safe and appropriate feeding [2]. Given regional differences in diet, clear guidance on complementary feeding is complex and difficult to distil into effective local and national policies [2]. Indeed, studies have shown that the same complementary feeding interventions have had varying results in different communities [3].

While breastfeeding in many parts of Africa extends beyond 1 year, breastfeeding is seldom exclusive to 6 months as recommended by the WHO [4]. Rather, food and water tend to be introduced relatively early, in parallel with continued breastfeeding. Infant feeding practices in South Africa differ from the rest of Africa, as breastfeeding rates are very low [5, 6] and use of commercial infant milk (hereafter referred to as *formula*) is normative.

There are wide-ranging implications for infant health in relation to w*hether* women breastfeed, *for how long,* and how *exclusively* [7]. However, these health implications are not straightforward. In low and middle income countries (LMIC) the acute consequences (primarily diarrhoea and related death) of suboptimum breastfeeding have been a priority in promoting breastfeeding. Globally, optimum breastfeeding could save as many as 823,000 lives annually [7]. However, in Cape Town there are relatively low rates of infant mortality as compared both to other parts of South Africa and other parts of Africa [8], which could be a result of the relatively good access to medical care. As such, acute concerns should be juxtaposed with the long-term developmental consequences of poor nutrition during gestation and the first 24 months of life. The consequences of both under nutrition and being overweight during this period are far-reaching, impacting not only acute illnesses and chronic disease but also healthy development and economic productivity [9, 10].

In recent years, several studies of breastfeeding in the context of migration have been published, although most take place in high-income countries (HIC). In a sample of 1875 migrant and non-migrant women, African migrants were most likely to breastfeed, and migrant women were almost twice as likely to be breastfeeding at 16 weeks postpartum in comparison to Canadian women [11]. However, this study did not measure the exclusivity of breastfeeding, which is important given relatively low exclusivity of breastfeeding in many parts of Africa, as well as the health implications of early introduction of both solid foods and formula. Nevertheless, Dennis et al.’s study suggested that African migrants retained some breastfeeding practices from their home countries, which is consistent with another large study set in Switzerland which suggested that breastfeeding rates are to some extent carried over from migrants’ countries of origin [12]. In a quantitative study of breastfeeding initiation in Australia, there was significant variation in the rates of breastfeeding initiation among Turkish (98% initiation), Australian (84%) and Vietnamese (75%) women [13]. A decline in migrants’ breastfeeding rates over time in the new country has been explained in terms of adopting the practices of the new country, as well as the lack of a supportive network available to mothers. For example, in the United Kingdom (UK), Pakistani and Bangladeshi immigrants felt that “everyone” (in the UK) bottle-feeds [14]. There is a need to understand how breastfeeding may differ for migrants in LMIC. For example, in South Africa, in cases where infants receive suboptimum feeding, there may be fewer supports available to migrants that those offered in HIC.

Breastfeeding practices in South Africa are likely to influence migrants in South Africa. In South Africa, rates of exclusive breastfeeding to 6 months of age are extremely low and estimated to be between 7% [5] and 10% [6], in comparison to exclusive breastfeeding rates in DR Congo (37%), Somalia (9%), and Zimbabwe (31%) [15]. Qualitative studies that focused on the perspectives of South African lactating mothers highlighted these very low rates of exclusive breastfeeding, lack of understanding of what ‘exclusive breastfeeding’ means, widespread perception of breastmilk inadequacy, and the early introduction of complementary foods [16–18]. Linked to this, stunting is the most common nutritional disorder amongst South African children, affecting 26.9% of boys and 25.9% of girls between the ages of 0 and 3 [19, 20]. While there is a clear policy agenda to improve the health of South African residents [21], there are also significant challenges to actually rolling out evidence-based interventions. Improving infant nutrition involves actively seeking to understand the experiences of populations including migrants who may be excluded from such interventions due to language, socioeconomic status, or legal status.

## Methods

Fieldwork for this study was conducted between February and October 2013. This article includes analyses of in-depth interviews, where individual descriptions were captured, and focus groups, which documented the ways that feeding was discussed in public (i.e. collective) settings. This paper is drawn from a broader study that included both maternal and infant nutrition amongst Congolese, Somali, and Zimbabweans living in Cape Town [22–24].

### Study setting

The study was conducted across greater Cape Town, where between 4% and 9% of residents were not born in South Africa [25]. The participants in this sample predominantly resided in rooms with their partner and children, and shared amenities with other families, either in a larger house or apartment, or in illegally subdivided warehouses.

### Study participants and sampling

Seventy-one individuals participated either in in-depth interviews (*n* = 23) or in a focus group (*n* = 48). Twenty-three Somali, Congolese, and Zimbabwean women were selected to include different migrant groups who could provide diverse insights. Participants were selected initially through local non-governmental organisations (NGOs) and then using a snowball approach. Participants (reported in more depth in a previous publication [22]) had typically arrived to Cape Town in the past 5 years, with a wide range of educational backgrounds. The interview inclusion criteria was women over the age of 18 pregnant or who had given birth in the last 2 years, and self-identified as Somali, Congolese (from the Democratic Republic of Congo, DRC), or Zimbabwean. Nine focus group discussions (*n* = 48) were held with adult Somali, Congolese, and Zimbabwean men (3 groups; *n* = 21) and women (6 groups; *n* = 27), segregated by country of origin and gender [22].

### Data collection

The lead author conducted all interviews and focus groups in English. A trained interpreter was used for two focus group discussions (22%), four Somali in-depth interviews and one Congolese in-depth interview, which were all conducted in both English and a second language (22% of in-depth interviews). In-depth interviews lasted between 45 min and 1.5 h, and were primarily conducted in participants’ homes. Focus group discussions took place in community centres and lasted between 1.5 h and 2 h.

For in-depth interviews, an interview guide included questions broadly related to experiences, meanings and understandings of maternal and infant nutrition. Specific lines of questioning related to breastfeeding included general breastfeeding questions (e.g. How have you fed your infant? How did you expect to feed your infant?), questions related to breastfeeding in the context of migration (e.g. How did your feeding choices compare to women back in [country of origin]?), conceptualisations of health in relation to infant feeding, and questions related to sources of support and information. Questions in focus groups broadly related to comparing experiences of nutrition for pregnant mothers and new babies in South Africa versus participants’ countries of origin (e.g. Could you talk about how people feed their babies in your community?).

Ethics approval was granted for this study from the University of Cape Town, Faculty of Health Sciences, Human Research Ethics Committee (Ref 009/2013). Participants’ interviewed were given R50 (equivalent to about one-third of a days’ wage for a domestic worker at the time of interviewing [US$5]). The interviewer obtained informed consent from all participants (including permission to record interviews) and clarified the opportunity to opt out at any time.

### Data analysis

All interviews and focus groups recordings were transcribed verbatim. Either a Somali or Congolese bilingual professional checked transcripts involving interpretation. On-going analysis took place throughout the research process in the form of a research diary, notes, and reflections.

Thematic analysis was used to generate a codebook and code all transcripts [26]. After initial immersion in the transcripts, potential codes were suggested as a way of categorizing and making meaning of the transcripts. Once no new categories of codes appeared, the codebook was considered complete, and coding commenced. All transcripts (focus group and in-depth interviews) were uploaded to the qualitative coding software Hyperresearch (Researchware Inc., 2009, Massachusetts, U.S.A.), to assist with coding, sorting, and data management.

## Results

While recalling a strong breastfeeding tradition in their home countries, cross-border migrants in this sample did not refer to “exclusive” breastfeeding or specific infant feeding recommendations or intentions. In this section, we explore the dominant discourse in terms of the three emerging themes from the findings related to infant feeding:

1. *Responsive rather than prescriptive*: The theme of being pragmatic and flexible in infant feeding decisions, responsive to a baby’s cues and weight gain. This is juxtaposed with *not* being prescriptive about medical recommendations.
2. *“A culture of formula”*: Feeding babies with commercial formula viewed as “normal” in the South African context. In contrast, limited formula feeding back home was largely described in terms of expense, rather than a preference for breast milk over formula milk.
3. *Work, stress and poor diet*: Low rates of breastfeeding were understood to be a result of work, general stress and poor diets - including household work and a lack of breastmilk supply.

### Responsive rather than prescriptive

Rather than describing a desire to follow the guidance of nursing staff or others, participants emphasized their flexibility and independence in making infant feeding decisions. Participants did not usually describe feeding goals, norms, or preferences in terms of either “cultural” norms or medically recommended ways to feed a baby. Rather, mothers described their feeding decisions in terms of whether or not their baby matched their expectation of “normal”:

For the feeding, they [Somalis] don’t have a month… it depends on the personal [person]. So some people start feeding their baby as early as 3 months, 4 months… Some don’t feed until after 1 year…

28-year-old Somali mother of two

When asked why she introduced food against medical recommendations, a participant felt she did so, “. . . because she [baby]would not stop crying. When I give her the food, she stop crying. If she [is] full, she sleep” (32-year-old Congolese mother of two). Thus breastfeeding tended to be exclusive only for as long as a mother perceived her child as growing and not crying “too much” (32-year-old Congolese mother of two). Some women saw breastfeeding as sufficient for small babies but not for babies with higher birth weights, or beyond 1 or 2 months of age. While breastfeeding was understood as the default feeding option at birth, solids and formula were quickly introduced as solutions to perceived problems such as in response to a baby’s growth, and in response to crying. When asked why breastmilk was no longer enough for her 2-month old, this respondent felt that:

…Because [baby’s name] is growing, then breastfeeding only is not enough. I felt the baby has grown up now, so I cannot satisfy her needs.

24-year-old pregnant Somali mother of one (interpreter present)

Participants’ described themselves as experimental in their approach to infant feeding:

It depends on how you feed the child. Like my firstborn‚ he was 3.8 (kg)‚ he was very big. And 2 days, when I give birth, he started to cry too much. Then, I was very young. I said, “what I should I do?” and I grew up with my auntie giving her kids mielie meal porridge and I… and so I take mielie meal and I put in the cup then I take water and…thin water‚ and I cook it. Then I give the child the porridge, he sleeps. So I just realized then‚ ok‚ my milk is not enough for him‚ so every time, like when we are going to sleep, I feed my child. Four or five spoons. He sleeps. He doesn’t cry.

Zimbabwean Women’s Focus Group

In the two quotes below, participants weighed medical advice against their experience, and felt that their experience affirmed their choice to depart from perceived recommendations:

. . . we were introducing solid foods. The doctors were not . . . they didn’t like it . . . they were discouraging us, but we, we used to find a way.

Zimbabwean men’s focus group

But I know it was not a good idea, with the boy, because nurses advise you to either breastfeed, or feed with formula‚ but I was doing both and *it worked*! The baby was healthy!

28-year-old Somali mother of 2

As described above, participants often spoke of medical advice, and seemed to have internalised clinic guidelines. However, the consensus was that introducing commercial infant formula and food did not have any downside. When asked whether there were any negative consequences of introducing foods at an early age, participants were unanimous:

All: No!

R2: 1 day you start to give the porridge, cereal . . . he gets fat . . . [] . . . you go you find he’s 10kg, next month you see he gets one more kg. [Laughter] so you see breastfeeding is maybe not enough for him. But if you give the porridge he may [grow quickly].

Zimbabwean women’s focus group

The value placed on having a fat baby contributed to the addition of formula at a young age. Weight gain was a highly subjective matter:

I heard a number of people talking about . . . advising people towards formula [. . .]. A child using formula will gain weight much quicker than a child who is breastfeeding. Some understand the importance of breastfeeding so they might continue breastfeeding, but they add formula, supplement with formula. Because to them when a child is not . . . when the degree of fatness is not fat enough, they freak out and they say . . . oh ok, my child is too slim what should I do what should I give him . . . they even ask in public . . . what did you give that child . . . he has nice body!

30-year-old Somali mother of 1

Thus the early addition of formula, water and solid food was voiced as a response to perceived problems. They were typically not expressed in terms of cultural norms, medical recommendations or breastfeeding intentions. Apart from rare exceptions, women did not usually describe why breastfeeding might be important, what benefits breast milk might provide the baby, or what harm might arise from formula feeding or the early introduction of solid foods.

### “A culture of formula”

The second theme related to the norm of commercial formula feeding in South Africa. Memories of breastfeeding back home were contrasted against descriptions of formula feeding in South Africa. Male migrant participants from all three countries described breastfeeding as the norm:

There is a difference that I see in South African culture when it comes in breastfeeding and Congolese culture. We believe in breastfeeding a lot because our children, even back home, our mothers, they breast feed more, you can see a child was up to the age of a year or a year and a half or 2 years still breastfeeding. But here, it’s like a culture of [formula] milk replacing natural breastfeeding milk

Congolese men’s group

The availability and affordability of formula in South Africa was one perceived benefit of living in Cape Town. While purchasing formula took up scarce resources, it was perceived as a necessity in Cape Town. In contrast, formula back home was considered prohibitively expensive. Rather than framing breastfeeding in terms of specific benefits, participants felt that the reason that women breastfed more in their countries of origin was due to the cost of formula feeding. Formula was presented as a status symbol:

They [friends] say ‘no I can’t breastfeed’. Me I didn’t breastfeed because the baby didn’t want . . . But the other people they do it like it’s proud they say ‘yes we got money I’m gonna buy formula’. . . ‘I’ve got money I’m gonna buy Nido . . . Nan’ [formula] you see? Other people they doing that.

30-year-old Congolese mother of 3

Formula feeding was also favoured for its observed benefits, primarily because it was perceived to extend infant sleep and help a child gain weight more rapidly than with breastmilk alone. The introduction of formula was often described in relation to having a sleeping, contented baby:

But then formula is a bit sustaining if you see if a baby finishes a whole bottle of formula. It’s going to sleep for quite a long time; but then breastfeeding in small amounts and the demand is like breastfeed now a few minutes later he wants to breastfeed again . . . So I think formula makes them more full.

27-year-old Zimbabwean mother of two

These sentiments seem to reinforce the overarching narrative of flexibility and pragmatism around infant feeding. However, this pragmatism favoured the introduction of both formula and complementary foods at an early age, due to the affordability of formula as well as the stressful circumstances of life in Cape Town. These stressful circumstances, and their relationship to breastfeeding, will be explored as the final theme, in the next section.

### Work, stress and poor diet

The overarching theme of stress and poor diet seemed to motivate participants’ perceptions of being responsive to their babies’ cues and navigating the norm of formula feeding in Cape Town. Underlying stressors were discussed in relation to the dominant perception of breastfeeding as impractical. It was not inevitable that breastfeeding was considered more difficult than other feeding choices: Indeed, back home breastfeeding was seen as entirely practical for “non-working” mothers. “Work” constituted one important stressor. Across all three migrant groups in the study, amongst respondents both formally employed or not, “work” was repeatedly described as a dominant influence on infant feeding. Even for migrants with formal refugee status, Cape Town was primarily described as a place of work, not a place of refuge.

Participants’ contrasted their own breastfeeding experiences with the breastfeeding practices of their mothers, and when doing so they contrasted their mothers’ role as “full-time housewife,” to their own roles as co-provider. Participants distanced themselves from the roles of their mothers’ and peers who defined themselves solely as mothers:

In Congo it’s easier [to breastfeed] because . . . [They are] not working they are just mother in the house . . . Sleeping . . . So they have that time . . .

28-year-old Congolese mother of three

Such memories affirmed the notion of breastfeeding as something one did when one did not have anything else to do. Yet while some women worked outside of the home, many participants did not. Participants from all three countries emphasized that a large number of relatives took on supportive roles in their countries of origin. For example, in Congo women described being able to ask nieces, cousins, or aunts to buy food, cook or clean on their behalf. In contrast, in South Africa errands of any kind were more complex and time consuming, and were considered to be work. For example, participants in Cape Town described having to travel on public transportation to get food, stand in long queues to transfer money, seek healthcare, or go on repeated trips to Home Affairs to appeal their asylum status were considered as work. Participants were also under significant financial stress, and unlike South African permanent residents or citizens, were usually ineligible for child grants. These stressors contributed to the sense that the urban environment was a place of work and hurry, and that these were incongruent with exclusive breastfeeding.

Women described the stress of life in a new city in relation to both lack of milk supply, as well as a baby’s refusal to breastfeed. Somali women raised the issue of low milk supply much more strongly than other migrant groups. While several women spoke about their infants refusing to breastfeed, this apparent refusal took place alongside the pain of unanticipated caesarean sections and lack of support during healing, the unintended consequences of the early initial introduction of formula, and the length of the queue at the clinic, which all factored into what was initially presented as a baby simply refusing breastmilk:

When I give the breast, he doesn’t take it. [. . .] They [hospital] tell me I have got to [breastfeed] him, I have to bring [the baby] back . . . they want to train the baby to adapt to breastfeeding, but because of the operation . . . [caesarean section] . . . I couldn’t do the queue and there, and so I didn’t go. So there was a problem with my son but I have also got difficulties‚ so I compromised . . . I was feeling the wounds and from the operation.

23-year-old Somali mother of 1 (Interpreter present)

Somali women consistently described their failure to breastfeed in terms of external forces beyond their control. These included physical illness, their infants’ refusal of one or both breasts, lack of milk supply - which was sometimes related to having a baby via caesarean section, but often because of perceived lack of weight gain or crying. This was contrasted with the religious standard of the Qu’ran, which Somali women highlighted as prescribing breastfeeding for 2 years. In the Somali men’s focus group discussion, participants highlighted that because women no longer breastfed, a typical 2-year age-gap had been greatly reduced, and Somali women in Cape Town were bearing more children as a result.

Stress in Cape Town was seen as inevitable given women’s status as migrants, and lack of milk supply was presented as similarly inevitable. In individual interviews, every Somali participant provided a reason that breastfeeding had been impossible or inappropriate from when their baby was 2 months or younger. While Somali women in focus group settings presented breastmilk supply as typically sufficient for a baby’s needs, the dominant narrative was around Somali women in Cape Town who lacked breastmilk supply because they “don’t eat enough” or “because of stress” (Somali women’s focus group). With the perspective of stress as a potential cause of low milk supply, formula feeding had become the acceptable default feeding option for all Somali mothers.

Participants’ flexibility and pragmatism overlaid with stress and busy-ness in Cape Town seemed to have unintended consequences. While women frequently had not intended to stop breastfeeding, introduction of formula or complementary foods quickly led to a cascade of events (low milk supply, bottle preference) that ultimately meant women stopped breastfeeding very early in their infants’ lives.

## Discussion

Participants’ descriptions of infant feeding departed from WHO recommendations at several junctures. Not unlike previous studies of populations who introduced solid foods early [27, 28], participants in this study had typically heard and understood official feeding guidelines, and made decisions on whether this advice made sense in their circumstances. This experimental and pragmatic approach implied self-awareness and comfort with their approaches to infant feeding. Feeding choices were thus neither framed in terms of medical recommendations, nor in terms of recommendations from other women, including elders.

Shaw and Wallace (2003) and others have studied the ways in which a community’s collective experience of food scarcity in the recent past makes women in that community more likely to favour excess food over risking that a child may be hungry [29]. Studies in HIC have presented the case that the ideal of a “fat baby” is associated with individuals of low socioeconomic status [30]. To some extent, explicitly in the Somali community, our findings affirmed this sentiment. The perceived need for food early in life may partly emerge out of migrants’ historical experience of food scarcity. Consistent with other studies of Somali experiences of war trauma [31, 32], Somali participants also shared a profound experience of stress and trauma. Participants reported on the embodiment of stress manifested in low milk supply, rather than discussing it openly or collectively. Stress is considered to be one of the primary ways that poor socioeconomic status increases levels of morbidity and mortality [33], and perhaps “low milk supply” and associated low rates of breastfeeding represents one mechanism for this association.

While expressing the losses that are wrought by migration and their tenuous circumstances in South Africa, participants’ descriptions of feeding their babies were imbued with self-confidence. In a previous paper, we described the linkages between social supports and the absence of elders in migrant communities [23]. Indeed, participants’ perspectives on their infant feeding practices are surely linked to mothers’ independence and relative social isolation, and to the absence of their own mothers. This confidence is a departure from at least some of the mainstream breastfeeding discourse. Literature that is focused on breastfeeding as a personal choice tends to juxtapose the strong intention to breastfeed against women’s actual experiences, which are sometimes in conflict [34, 35]. Noting the relationship between breastfeeding self-efficacy and breastfeeding duration amongst Japanese mothers, one study argues for education and reassurance by medical providers of adequate milk supply [35]. This and other studies of breastfeeding tend to be premised on Bandura’s theory of self-efficacy [36], which assumes that women want to be effective at breastfeeding. However, this study seemed to suggest that while women were generally familiar with official feeding guidelines, their self-perceived effectiveness at breastfeeding was not necessarily the primary factor in guiding their infant feeding choices. Moreover, while notions of the ideal breastfeeding mother loom large in many western settings [37], participants in this sample did not express these ideals, nor the intention to breastfeed a certain amount of time, nor the sentiment of guilt over infant feeding decisions. Rather, being a competent mother seemed to be considered in terms of having a healthy, growing baby, and in providing for the family’s physical needs by running errands or working. Participants also had pressing concerns related to housing, legal documentation, work, and responsibilities to family remaining back home, thus while a healthy baby was important, following specific feeding recommendations were of low priority.

Given difficult circumstances after migration, the perceived norm of formula feeding in Cape Town, and its perceived relative affordability, seemed to affirm something positive about life in a new city. Participants referred to Cape Town as a wealthy city; a place for earning enough money to support immediate families and often also their extended families back home. Yet it was also a place where these same migrants were often undocumented, unemployed and living in crowded and substandard housing. In the midst of these contradictions and despite its cost, formula feeding affirmed the former, more successful, identity.

The categorization of Cape Town as a place of work was also part of a broader self-identification as co-provider rather than primarily mother or wife. As such, living in Cape Town was itself “work”, even for the majority of women who never earned their own income. Such self-identification also had important implications for gender roles, and the ways that gender roles intersect with breastfeeding [38]. Given that women’s work has been consistently under-valued in multiple settings around the world [39], it is notable that participants’ definitions of work were revealed as broad, encompassing the work of the home and the act of living in a foreign country. “Work” is a significant variable described in many studies from HICs impacting breastfeeding; in studies in multiple countries it may even be the most important variable [40, 41]. Life in Cape Town constituted “work” in several ways: Rentals were tenuous, families lacked private space, had fears over their safety, and participants perceived friends and family to be “busy”. Pressing errands undermined women’s ability to breastfeed, as these trips were uncomfortable, difficult or even impossible to combine with regular breastfeeding.

### Study limitations

This study focused on a small group of participants living in Cape Town, which is particular in some ways: it reflects LMIC-LMIC migration. Cape Town also has good services relative to other cities in South Africa and Africa. Participants’ use of formula reflects both the urban context and at least some access to funds. The use of interpretation, and conducting at least some interviews in a second language, presented some limitations to the depth of the study and the conclusions that can be drawn from our findings. That said, third party professionals checked the quality of interpretation and discussed the transcripts at length with the primary author. This allows us to be confident of the overarching themes, particularly given the relatively low percentage of data that involved language interpretation.

## Conclusion

This Cape Town based study of cross-border migrants from DR Congo, Zimbabwe and Somalia found that while women expressed a desire to breastfeed, and typically breastfed in the first weeks after birth, breastfeeding was not prioritised. This was not entirely unexpected, given the norm of early complementary feeding in women’s countries of origin. The lack of prioritisation of breastfeeding is also set against the perceived norm of formula feeding in Cape Town and significant life stressors.

The practical challenges of breastfeeding described by cross-border migrant women reflect one way in which socio-economic and health inequalities may currently be perpetuated for marginalised populations. These upstream barriers to breastfeeding are beyond the circumscribed role of most public health interventions. Nevertheless, the barriers to breastfeeding amongst migrant women add to the literature relating the complexity of socioeconomic inequality to health. Moreover, while the contextual factors that make exclusive breastfeeding impractical seem intractable, novel approaches may help to make more extended, or more exclusive breastfeeding practical. For example, one NGO in the greater Cape Town area (*Philani*) specialises in offering intensive mothering support to the urban poor, targeting issues of social isolation and other overarching challenges to caring for babies. Clinics and NGOs should also consider focusing on social isolation amongst migrants or on how to feed an infant while working or running a household in an urban LMIC context.

Sub-optimal breastfeeding and complementary feeding practices are a complex mechanism through which long-term health and developmental inequalities may be perpetuated in marginalised migrant populations. Indeed, the reasons for early introduction of both formula and solid foods were complex, and in most cases participants were aware of medical recommendations. Rather than emphasizing specific breastfeeding intentions, participants favoured an approach that reacted to their perceptions of a baby’s changing needs. Participants also did not associate the early addition of formula and complementary foods with declining breastmilk supply - that is, they seemed less aware of the ways that breastfeeding supply changes in response to increased and decreased feeding. There was also little discussion by participants related to the long-term health benefits of breastfeeding. Increased knowledge of these long-term benefits, as well as an understanding of the mechanics of increasing breastmilk supply, could provide a healthy reframing of infant feeding. Targeted education could shift attention away from short-term health and weight gain. Clinic-led antenatal interventions providing specific information regarding breastfeeding supply and highlighting the long-term, “invisible” benefits of breastfeeding may prove more effective in improving infant feeding than more general breastfeeding education.

## Abbreviations

HIC: High income countries
LMIC: Low and middle-income countries
NGO: Non-governmental organisation
